# One-dimensional diamondoid polyaniline-like nanothreads from compressed crystal aniline

**DOI:** 10.1039/c7sc03445h

**Published:** 2017-10-18

**Authors:** Marcelo M. Nobrega, Erico Teixeira-Neto, Andrew B. Cairns, Marcia L. A. Temperini, Roberto Bini

**Affiliations:** a Departamento de Química Fundamental , Instituto de Química da Universidade de São Paulo (USP) , CP 26077-CEP 05513-970-São Paulo , SP , Brazil . Email: nobregam@iq.usp.br ; Fax: +55 11 3091 3890 ; Tel: +55 11 3091 3890; b LENS , European Laboratory for Nonlinear Spectroscopy , Via Nello Carrara 1, 50019 Sesto Fiorentino (FI) , Italy; c Dipartimento di Chimica “Ugo Schiff” dell’Università degli Studi di Firenze , Via della Lastruccia 3, 50019 Sesto Fiorentino (FI) , Italy; d ICCOM-CNR , Institute of Chemistry of OrganoMetallic Compounds , National Research Council of Italy , Via Madonna del Piano 10, I-50019 Sesto Fiorentino , Firenze , Italy; e European Synchrotron Radiation Facility , 71 Avenue des Martyrs , 38043 Grenoble , France; f Brazilian Nanotechnology National Laboratory (LNNano) , Brazilian Center for Research in Energy and Materials (CNPEM) , Campinas, Sao Paulo , Zip Code 13083-970 , Brazil

## Abstract

Low-dimensional nanomaterials such as highly ordered polyaniline (PANI) have attracted considerable interest due to their expected extraordinary electronic and optoelectronic properties. In spite of several attempts, the attainment of atomically well-ordered PANI is a long-standing challenge. Pressure-induced polymerization of aromatic molecules in the crystal phase has been demonstrated as a practicable route for the synthesis of highly ordered polymers but this approach has never been tested to produce PANI. Here we show the synthesis of diamondoid polyaniline-like nanothreads at 33 GPa and 550 K by compressing aniline in crystal phase-II. Infrared spectroscopy, transmission electron microscopy, X-ray diffraction data, and DFT calculations support the formation of this totally new polyaniline-like nanothread. The NH_2_-enriched carbon nanothread combines the outstanding mechanical properties of carbon nanotubes with the versatility of NH_2_ groups decorating the exterior of the nanothreads representing potential active sites for doping and as linkers for molecules with biological interest and inorganic nanostructures. The synergy of all of these properties emphasizes the strong potential of this material to be applied in a broad range of areas, from chemistry to materials engineering.

## Abstract 2

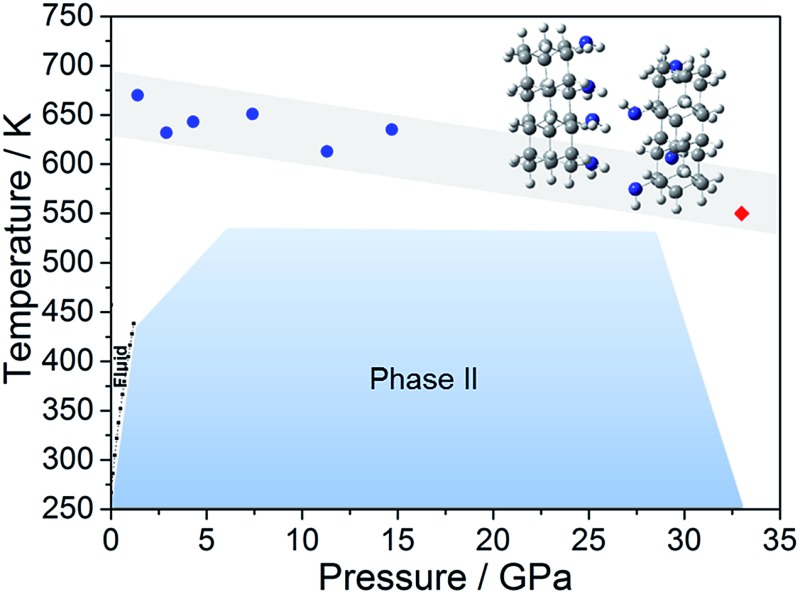
One-dimensional diamondoid polyaniline-like nanothreads combine the outstanding mechanical properties of carbon nanotubes with the versatility of NH_2_ groups.

## Introduction

The obtainment of highly-ordered low-dimensional polyaniline (PANI) is a long-standing challenge of great interest for a large part of the scientific community. In fact, PANI is expected to present extraordinary electronic and optoelectronic properties, exhibiting strong potential in several fields, covering from fundamental chemistry to applied materials science.^1^–^3^ Recently, synthesis of a real 2D PANI framework has been reported,^1^ nevertheless, due to the complexity in the mechanism of aniline polymerization, full control of the PANI structure at the atomic scale has not yet been achieved.^4^ In this framework, pressure-induced polymerization of aniline in the solid-state could represent an attractive alternative route for obtaining ordered PANI.

Solid-state chemistry induced at high-pressure and high-temperature has been successfully used in the search for new and fascinating materials such as confined polymers and extended amorphous networks.^5^–^9^ A possible advantage of these reactions is represented by the topochemical constraints posed by the crystal that can give rise to products closely recalling the symmetry of the molecular crystal from which it is formed.^6^,^10^,^11^ In some cases the pressure and temperature conditions required for the synthesis are such that they can be easily scaled up, thus representing a ‘green’ method appealing to industrial chemical synthesis, since the use of additional and polluting compounds such as initiators, catalysts and solvents is avoided. For example, high density polyethylene was successfully synthesized at high-pressure and the reaction conditions are completely accessible to the current industrial technology.^12^

Recently the formation of a 1D, highly ordered, saturated nanomaterial with a diamond-like local structure was reported after the compression of benzene up to 20 GPa.^6^ Such diamondoids are “cage-like” structures consisting of fused cyclohexane rings that exhibit complex structures and geometries.^13^ With their unique and stable molecular structures, diamondoids and their derivatives are a new generation of novel and durable materials and devices for nanotechnology applications.^13^ An aniline-rich and/or amino-rich diamondoid-derived nanomaterial could combine the atomic-level uniformity of diamondoid materials and inherit PANI properties, that can be tailored by changing the dimensionality of the nanostructures,^14^ and is expected to be a potentially relevant technological material. Previous experiments on the high-pressure behaviour of aniline have shown a remarkable stability of the monomer under quite high pressure and temperature conditions possibly due to strong directional H-bonds.^15^

Here, for the first time to our knowledge, we have induced the transformation of aniline to a pale yellow-brownish recoverable one-dimensional diamondoid-like polyaniline by compressing aniline to 33 GPa and heating to 550 K. Infrared spectroscopy, transmission electron microscopy (TEM), X-ray diffraction data, and density functional theory (DFT) calculations support the formation of this totally new polyaniline nanothread.

## Results and discussion

Literature data have showed that aniline crystal phase-II is anomalously chemically stable in a broad *P* and *T* range when compared to other aromatic molecules.^6^,^20^–^22^ This occurrence is likely related to the longer C–C contacts between adjacent molecules in aniline, a consequence of the strong and directional H-bonds along the *c*-axis.^15^ In order to estimate the *P*–*T* reactivity threshold of aniline at several *P* and *T* conditions, we have employed a model accounting for thermal displacements already used for *s*-triazine^5^ and benzene^10^ crystals adopting the critical distance, 2.5–2.6 Å between the closest intermolecular C–C contacts found in these cases. As reported in ref. 10, the maximum instantaneous linear displacement from the equilibrium position (2*a*_m_) can be estimated as 3*σ*, accounting for more than 99% of the displacement amplitudes, where *σ*^2^ is classically given by:1
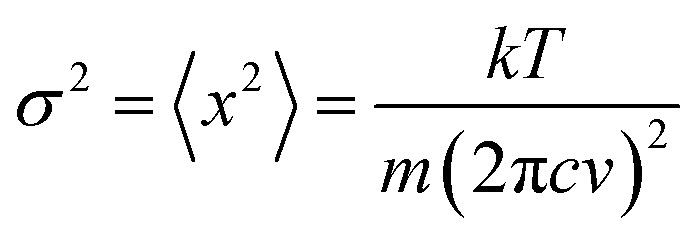
where *k* is the Boltzmann constant, *T* the temperature, *m* the molecule mass, and *ν* the frequency (cm^–1^) of the phonon mode. The pressure evolution of the density of states (DoS) of the lattice phonon modes was estimated using the relationship *F*_*P*_ = *F*_*P*_0__(*V*_0_/*V*_*P*_)^*γ*^, where *F*_*P*_0__ is a representative phonon frequency at *P* = 0 GPa (62 cm^–1^), *V*_0_ and *V*_*P*_ are the volumes at *P* = 0 GPa and at a given pressure (*P*),^15^ and *γ* is the Gruneisen parameter. A *γ* value of 1.5 accounts for a regular linear evolution of the instability boundary.

**Fig. 1:**
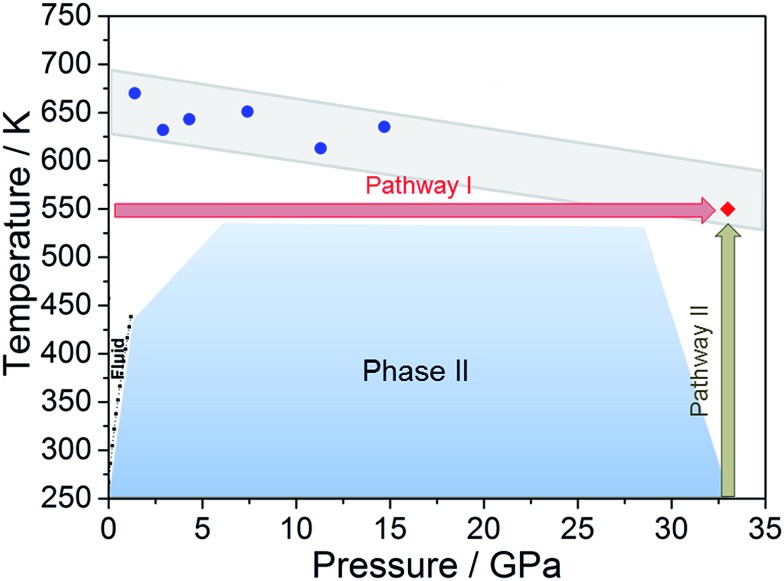
The blue area represents the stability *P*–*T* range of aniline explored experimentally (see ref. 15). The full blue circles represent the estimated reactivity thresholds of aniline phase-II, according to Table 1, while the gray area is an estimate of the uncertainty of this determination. The full red diamond represents the induced reactivity of aniline phase-II at 33 GPa and 550 K. The black squares linked by a line represent the reported liquid–solid transition boundary from ref. 23. *P*–*T* pathways followed to trigger the reactivity in phase-II aniline are also indicated.

In Table 1 we report, for given pressures, the estimated thermal corrections according to eqn (1) and the corresponding temperature thresholds. Our calculations suggest that aniline is stable in a broad *P*–*T* range, see Fig. 1, overcoming the *P*–*T* stability of other comparable molecules such as benzene,^10^*s*-triazine^5^ and pyridine.^20^ Because of the approximations used in this calculation, the reactivity threshold is not reported as a line (see gray area in Fig. 1) to account for the uncertainty in the *P* and *T* values.

**Table 1:** Estimated thermal corrections and threshold temperatures for given *P* conditions in which the nearest neighbor C–C was equal to 2.6 Å

*P* (GPa)	Structural *d* (Å)	Thermal 2*a*_m_ (Å)	Estimated threshold *T* (K)
1.4	3.585	0.985	**670**
2.9	3.526	0.926	**632**
4.3	3.450	0.850	**643**
7.4	3.353	0.753	**651**
11.3	3.278	0.678	**613**
14.7	3.266	0.666	**635**

After having estimated the chemical instability boundary for aniline, an isothermal compression at 550 K was performed (Fig. 1 – pathway I); a temperature that should correspond to a reaction onset between 30 and 35 GPa. This expectation, and consequently the previously adopted assumptions, were confirmed by the experiment. Indeed when the pressure was slightly lower than 33 GPa, aniline’s spectral features begin to weaken significantly, suggesting that the aniline became unstable under these *P* and *T* conditions.

**Fig. 2:**
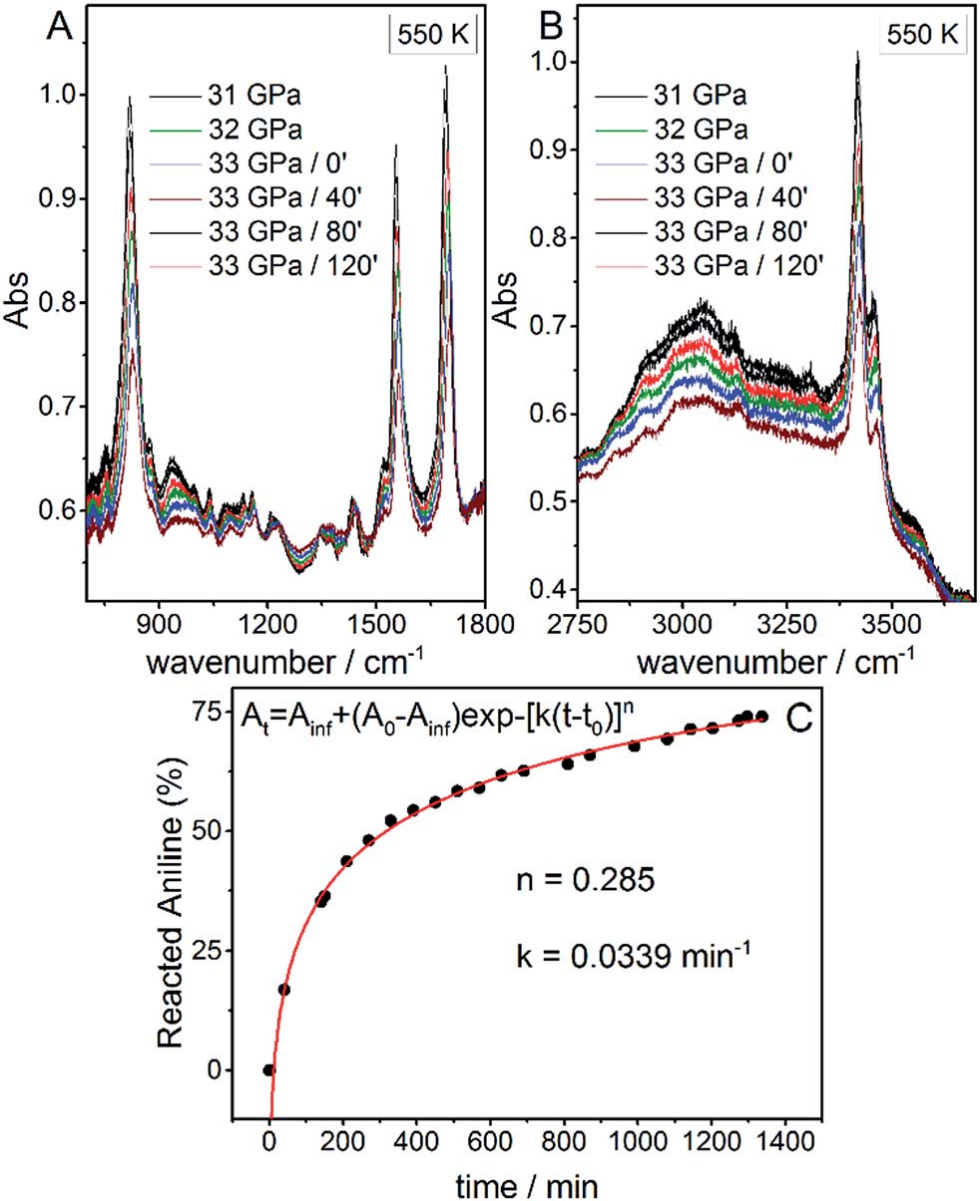
Infrared absorption spectra of aniline phase-II at 550 K and pressures ranging from 31 to 33 GPa. (A) 750–1800 cm^–1^ spectral range and (B) 2750–3800 cm^–1^ spectral range. After reaching 33 GPa, a significant decrease in the intensity of aniline spectral features is observed, indicating the occurrence of a chemical reaction. (C) A kinetic curve representing the time evolution of aniline consumption at 33 GPa and 550 K. The full red line corresponds to the fit performed using eqn (2).

Once the *P* and *T* values to induce aniline’s reactivity had been identified, another fresh sample was prepared, however, in this case, a KBr pellet was used to reduce the optical path avoiding saturation of the absorption bands and allowing the kinetic study of the reaction. The sample was first compressed up to 33 GPa and then heated to 550 K (Fig. 1 – pathway II). After reaching the reaction onset, the kinetics was followed over about 24 h. The observation of the reaction at the same *P*–*T* conditions rules out any effect on the reactivity of the *P*–*T* path followed and of the salt substrate. The initial four spectra measured during the kinetics study are presented in Fig. 2A and B. The amount of reacted aniline was determined by the absorbance of the band at ∼830 cm^–1^, relative to the C–H out-of-plane bending mode.^24^ The absorption pattern was fitted using a Voigt profile and the total absorbance, the area of the band, was taken as a measurement of the amount of residual aniline during the evolution of the reaction. The data were analyzed with the Avrami model.^25^–^27^ A fitting equation derived from ref. 29, can be written as

2 *A*_*t*_ = *A*_inf_ + (*A*_0_ – *A*_inf_)exp –[*k*(*t* – *t*_0_)]^*n*^

where *A*_0_, *A*_*t*_, and *A*_inf_ are the integrated absorptions of aniline at the beginning of the reaction (*t*_0_), after a time *t* and at the equilibrium, respectively; *n* is a parameter that accounts for the growth dimensionality for a given nucleation law and *k* is the rate constant. The kinetic curve was nicely reproduced using the rate constant *k* and the *n* parameter reported in Fig. 2C. The *n* parameter can provide insight about the microscopic evolution of the reaction once it is related to the growth geometry and nucleation rate. An *n* value smaller than 1 unambiguously indicates unidimensional growth. In our case, the *n* parameter obtained was equal to 0.28, clearly indicating a product propagating with 1D growth. Similar *n* values were already reported in the pressure induced unidimensional polymerization of acetylene^28^ and ethylene.^29^

Once no further spectral modifications could be appreciated, the sample was first brought back to ambient temperature and then the pressure was slowly released. According to Fig. 2C, a certain amount of unreacted aniline, about 25%, was still present suggesting that lattice defects can prevent the 1D reaction propagation. Upon releasing the pressure, the intensity of all of the aniline bands decreased indicating that the reaction proceeds during the downstroke.

**Fig. 3:**
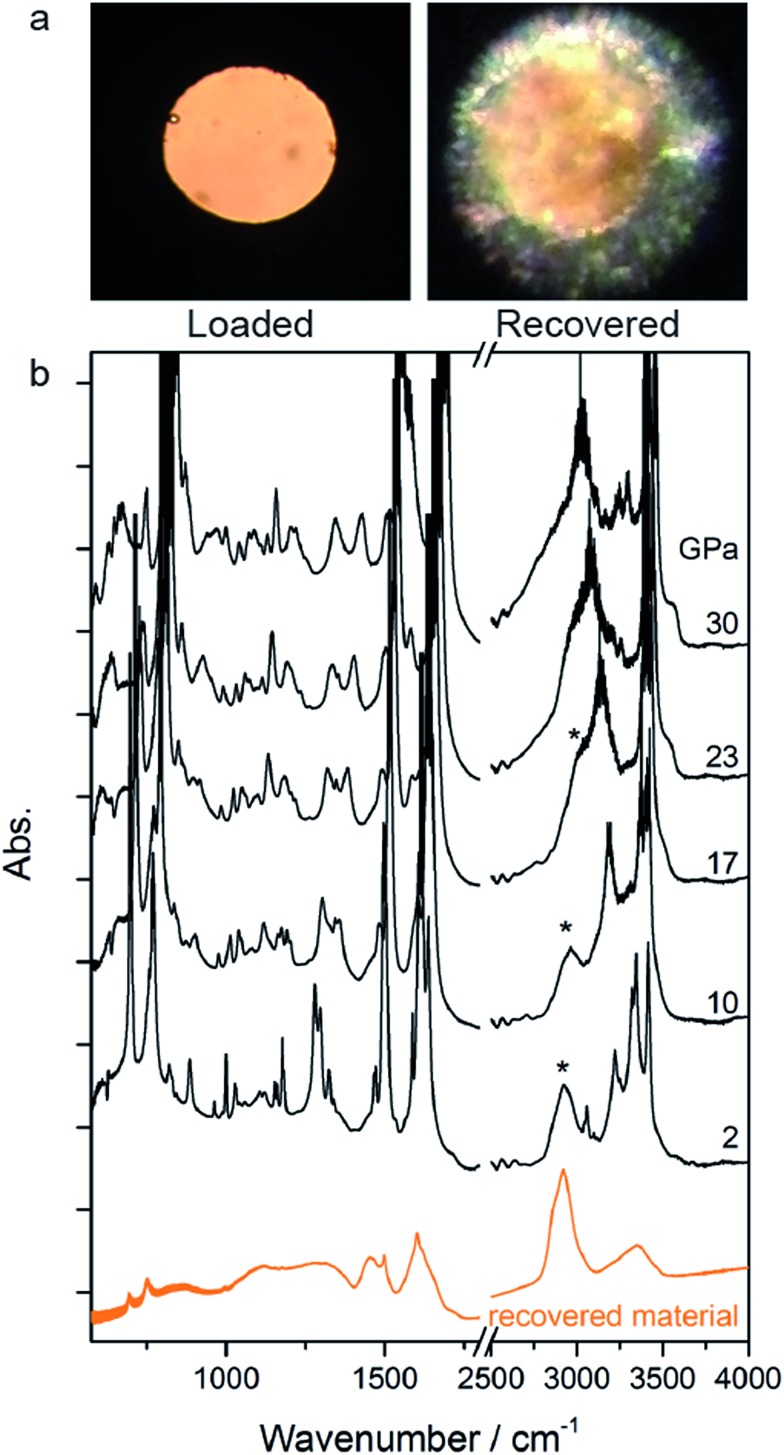
(a) Optical images of the loaded aniline and the recovered pale yellow/brownish material after decompressing and opening the cell. (b) Infrared absorption spectra for selected pressures recorded along the decompression cycle at 298 K and infrared spectrum of the recovered material, lower trace.

The rather homogeneous pale yellow/brownish material synthesized at high *P* and *T* and recovered at ambient conditions is shown in Fig. 3a. The infrared absorption spectrum of this material (Fig. 3b) resembles that of the product recovered from the high pressure reactivity of benzene thus suggesting a saturated material containing N–H bonds.^21^

Recently, a 1D, highly ordered, saturated nanomaterial with a local diamond-like structure was recovered after a controlled compression–decompression cycle of benzene up to 20 GPa.^6^,^30^ The product, characterized by a wealth of techniques like bright-field transmission electron microscopy and synchrotron X-ray diffraction, differed substantially from the amorphous material reported in previous reports,^10^,^21^ consisting of a tubular or thread-like structure, which was also supported by first principle calculations.^6^,^30^

**Fig. 4:**
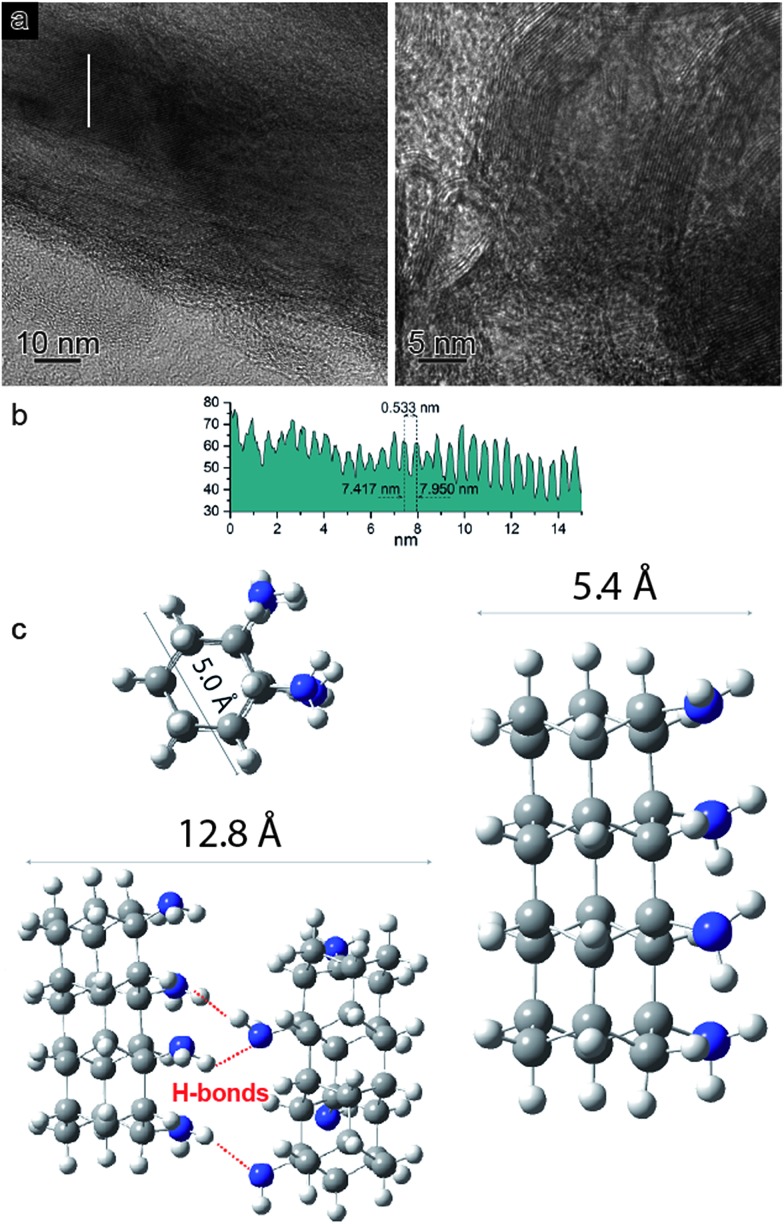
(a) BF-TEM images at two different magnifications (see inset scale bars) of the recovered material presenting striations spaced at ∼4 to 5.1 Å and extending in the longitudinal dimension tens of nanometers. (b) A line profile measured along the white line in the low magnification BF-TEM image (a – left). (c) Views of the optimized DFT geometry structure of the one-dimensional aniline-derived nanothreads with relative dimensions.

In view of the striking similarities with the benzene reactivity and the intriguing results of the Avrami model, suggesting a material characterized by 1D growth, the product was morphologically characterized using multiple techniques. The recovered material was mechanically removed from the gasket with a needle and placed directly onto the surface of a standard transmission electron microscope (TEM) copper grid. The collected multiple high-resolution TEM images at two different magnifications (Fig. 4a) exhibit parallel striations, suggesting the formation of threads or tubes. The line profile measured along the white line in the low magnification BF-TEM image (Fig. 4a – left) presented in Fig. 4b shows that the striations are ∼5.5 Å apart. The high magnification BF-TEM image (Fig. 4a – right) evidenced the presence of long-range 1D parallel striations spaced at 4.0–5.1 Å and sizing tens of nanometers long. These values can be compared to the 1-D nanothreads obtained from benzene which were characterized by a distance between packed threads of ∼6.4 Å.^30^ The regions where the linear threads bend, as visible in the image with higher magnification (Fig. 4a – right), are likely related to crystal boundaries or dislocations of the starting aniline crystals. It should also be remarked that the nanothreads develop along a specific aniline crystal direction, namely the *a*-axis (see following discussion), which should therefore lie in the image plane to make the threads observable. The latter issue can account for the regions where apparently no threads are present.

In order to support the spectroscopic and morphological characterizations, quantum chemical calculations were performed using DFT to model a residue of a 1-D polyaniline-like nanothread structure consisting of 4-fused aniline molecules in which the sp^2^ carbons of the rings were converted into sp^3^ carbons by forming covalent bonds that develop between the rings. Views of the optimized geometry of a segment that accounts for the aniline-derived nanothread with the relative dimensions are presented in Fig. 4c. According to the DFT optimized geometry, the diameter dimensions expected for an aniline-derived nanothread are in the order comprised between 5.0 and 5.4 Å, which is in striking agreement with the parallel striations observed in the TEM images.

**Fig. 5:**
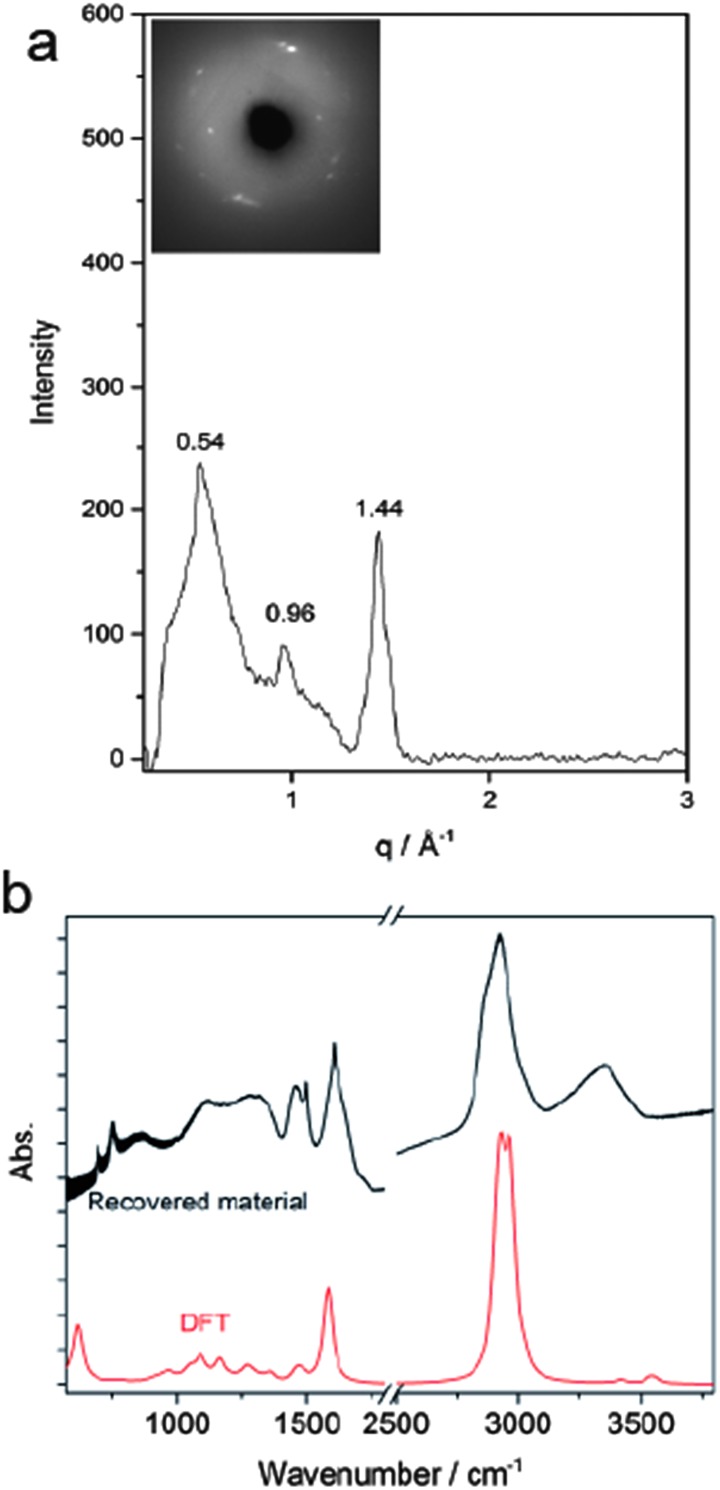
(a) Bragg peaks in the total X-ray scattering structure. The inset shows the original 2D diffraction image of the recovered material. (b) Upper black trace: IR absorption spectra measured at ambient conditions of the recovered sample removed from the DAC. Lower red trace: calculated IR spectra for the 1D aniline-derived nanothreads. Band shapes were modelled using Gaussian functions with a bandwidth of 20 cm^–1^.

According to Thess *et al.*^31^ a 2D triangular lattice is characterized by a group of peaks in the low-Q region: a strong peak around 0.44 Å^–1^ followed by four weaker peaks up to 1.8 Å^–1^. Angle dispersive X-ray diffraction measurement on the recovered material (Fig. 5a) notably agrees with the mentioned characterization, suggesting the formation of a triangular structure with an *a* = 13.3 Å, in the middle range of the lattice values reported for benzene-derived nanothreads (∼6.4 Å)^6^ and fullerene single-wall carbon nanotubes (17 Å).^31^ The lattice constant is two and half times the value of the nanothread diameter and almost double that of benzene derived nanothreads, which can suggest that the molecular orientation and the hydrogen bond network in the crystal strongly influence the reaction for aniline and the product characteristics. Crystal aniline presents a peculiar H-bond arrangement connecting the NH_2_ groups of the nearest neighbor molecules and developing along the *a*-axis.^15^,^32^ The presence of such strong interactions in the crystal prevents the participation of the NH_2_ groups in the reaction and favors the remarkably anisotropic compression along the direction more suitable for inter-ring interaction. The natural conclusion is that these constraints selectively drive the inter-ring formation of C–C bonds, with the consequent C hybridization change from sp^2^ to sp^3^, along the *a*-axis. Therefore, double nanothreads form along this direction interacting through H-bonds and having a size of ∼12.8 Å (Fig. 4c), resulting in an expanded 2D triangular lattice when compared to benzene. This is in excellent agreement with the lattice parameter (*a* = 13.3 Å) derived from the angle dispersive X-ray diffraction pattern.

Finally, the structure of the one-dimensional polyaniline nanothread with a local diamondoid-like geometry was optimized and its vibrational spectrum was calculated by quantum chemical calculations using density functional theory (DFT). Two views of the optimized nanothread geometry and one view of the double nanothread interacting through the H-bond region are presented in Fig. 4c. DFT calculations strongly support the formation of one-dimensional aniline-derived nanothreads with the structural parameters and the calculated infrared spectrum being in great accordance with the experimental data. Both IR spectra, calculated and measured, are dominated by the C–H stretching modes of sp^3^ hybridized carbon atoms at 2900 cm^–1^ and by the bending N–H modes at 1575 cm^–1^ (Fig. 5b) thus providing solid evidence for the formation of a one-dimensional aniline-derived nanothread.

The properties of different functionalized diamond nanothreads have been recently computed by DFT calculations.^33^ These materials correspond to the possible products of high-pressure transformations of both functionalized benzene and heteroaromatic rings. Interestingly, the functionalized diamond nanothreads maintain the mechanical properties of the pristine material but offer, depending on the functional group and its spatial distribution, the possibility of tuning the band gap. According to these predictions the NH_2_-enriched carbon polyaniline-like nanothread is expected to present a band gap in the order of 3.5 eV, an essentially insulating material due to the intrinsic carbon sp^3^ character, an ideal strength of ∼14.8 nN, a Young’s modulus of 163 nN and a fracture strain (*ε*_max_) of ∼0.16.^33^ Moreover, the synergic effect between these remarkable mechanical properties with the versatility of the NH_2_ groups decorating the exterior of these nanothreads representing potential active sites for doping and as linkers for molecules with biological interest and inorganic nanostructures, must be taken into account.

## Methods

Aniline (C_6_H_5_NH_2_, Merck) was distilled under reduced pressure prior to use and was loaded into a MDAC (membrane diamond anvil cell) equipped with IIa type diamonds and a rhenium gasket where a hole with an initial diameter of 150 μm was drilled and used as a sample chamber. In order to reduce the strong IR absorption of the sample, the optical path was reduced by pressing KBr into the sample chamber producing a pellet whose surface was successively scratched. Afterwards, liquid aniline and a ruby chip were added above the KBr pellet resulting in a sample thickness ranging from 10 to 20 μm. High-temperature experiments were performed using the resistively heated MDAC. The temperature was measured with an accuracy of ±0.1 K by a K-type thermocouple placed close to the diamonds. FT-IR absorption measurements were recorded with an instrumental resolution of 1 cm^–1^ using a Bruker-IFS 120 HR spectrometer modified for high-pressure measurements.^16^ The ruby fluorescence was excited using a few milliwatts of a 532 nm laser line from a Nd:YAG laser source.

Angle-dispersive X-ray diffraction (ADXRD) experiments were performed at the ESRF high-pressure beamline ID27 using monochromatic X-ray radiation of wavelength *λ* = 0.3738 Å, a MARCCD 165 detector positioned at 146 mm from the sample, as calibrated with a CeO_2_ standard. The focal spot (fwhm) of the beam was ∼3 μm. The 2D diffraction patterns were integrated using DIOPTAS; manual background subtraction was done in Fityk.

Bright-field imaging (BF-TEM) was performed using an FEI Titan Themis 60-300 transmission electron microscope operated at an 80 kV accelerating voltage. The sample was removed from the gasket with a needle directly onto the surface of a standard TEM copper grid.

Quantum chemistry calculations were performed using the Gaussian03 package^17^ to obtain optimized structures and vibrational frequencies for the one-dimensional diamondoid polyaniline-like model. Density functional theory (DFT) using the Becke’s three-parameter hybrid exchange functional and Lee–Yang–Parr correlation functional (B3LYP)^18^,^19^ and 6-311++G(d,p) basis set was used. No imaginary vibrational frequencies were obtained indicating that the vacuum geometries were at the minimum of the potential surface.

## Conclusions

The reactivity of aniline was induced by compressing the crystal phase-II above 30 GPa at temperatures in excess of 500 K. The onset of the reaction nicely agrees with the estimate based on a simple model accounting for thermal displacements. The reaction kinetics sharply indicates the formation of a 1D product. The latter was recovered at ambient conditions and characterized by transmission electron microscopy, X-ray diffraction and FTIR spectroscopy which provided evidence of the formation of a totally new diamondoid polyaniline-like nanothread. Inter-ring bonds give rise to a fully sp^3^ hybridized structure forming double nanothreads with diameters of approximately 12.8 Å and arranged in a 2D triangular lattice with an *a* parameter almost double that in benzene derived nanothreads. The reaction is strongly influenced by NH_2_ groups that, although not participating in the reaction, favor, through the H-bonding arrangement, anisotropic compression along the direction more suitable for the inter-ring interaction. In addition, the NH_2_ groups decorate the exterior of these nanothreads representing potential active sites for doping and as linkers for molecules with biological interest and inorganic nanostructures. The combination of these properties with a great Young’s modulus emphasizes the strong potential of this material to be applied in a broad range of areas, from chemistry to materials engineering.

## Conflicts of interest

There are no conflicts to declare.
